# Interface Behavior and Corrosion Study of MgO-Based Refractory Materials in Molten Pharmaceutical Waste Salts

**DOI:** 10.3390/ma19102057

**Published:** 2026-05-14

**Authors:** Qinhao Yang, Feng He, Weiwei Cheng, Manman Gao, Junlin Xie

**Affiliations:** 1 School of Materials Science and Engineering, Beijing University of Technology, Beijing 100124, China; yangqinhao@emails.bjut.edu.cn (Q.Y.); ischengweiwei@163.com (W.C.); 13791759627@163.com (M.G.); xjl09532@bjut.edu.cn (J.X.); 2 State Key Laboratory of Materials Low-Carbon Recycling, Beijing 100124, China

**Keywords:** pharmaceutical waste salt, MgO-based refractory materials, interface behavior, thermodynamic calculation, anti-corrosion mechanism

## Abstract

This study addresses the corrosion problem of refractory materials during high-temperature molten treatment of pharmaceutical waste salt, and systematically investigates the interface behavior and corrosion mechanism of MgO-based refractory materials in simulated pharmaceutical waste salt (65 wt% NaCl-30 wt% Na_2_SO_4_-5 wt% CaCO_3_). Through sessile drop wetting infiltration experiments, static corrosion tests (950 °C and 1150 °C/48 h), combined with SEM-EDS, XRD characterization, and FactSage thermodynamic calculations, the corrosion resistance of high-purity MgO phase (HM-97) refractory materials and magnesium–aluminum spinel composite phase (MA-85) refractory materials was compared and analyzed. The results show that due to the fine periclase grains and rich grain boundaries, the molten salt infiltration rate of HM-97 material in the 644–800 °C range is significantly higher than that of MA-85. After corrosion at 950 °C, HM-97 and MA-85 formed 47 μm and 53 μm transition layers respectively, and the HM-97 surface generated Ca_3_Mg(SiO_4_)_2_ phase leading to uneven corrosion morphology. At 1150 °C, HM-97 produced long cracks and the transition layer thickness remained almost unchanged due to dissolution, while MA-85 formed an approximately 72 μm transition layer and a dense metamorphic layer. Phase analysis and thermodynamic calculations suggest that the MgAl_2_O_4_ phase in MA-85 is likely stable at high temperatures, which appears to effectively prevent molten salt infiltration and contribute to forming a protective metamorphic layer, thereby potentially enhancing the material’s corrosion resistance. The MgAl_2_O_4_ phase is proposed to improve the service performance of MgO-based refractory materials in the molten pharmaceutical waste salt environment.

## 1. Introduction

With the rapid development of the fine chemical industries [1,2], pharmaceutical [3,4], pesticide [5,6], and other industries, the volume of high-salinity organic waste has surged dramatically, posing multiple challenges in its disposal. The waste salts typically contain inorganic components such as sodium chloride (NaCl), sodium sulfate (Na_2_SO_4_), potassium chloride (KCl), and potassium sulfate (K_2_SO_4_), along with persistent organic toxins that exhibit poor biodegradability [1,7]. Currently, landfill disposal remains the predominant method for handling such waste salts. However, due to their high water solubility, inadequate treatment of these materials may result in soil contamination through aqueous infiltration, thereby posing significant environmental risks [8]. Great attention has been paid to how to treat such waste salts and realize their resource-oriented utilization. China generates over 20 million tons of industrial waste salts annually [9], and the government has actively taken measures to encourage relevant enterprises to strengthen the resource-oriented treatment of these waste salts. Extensive research has been conducted on the resource utilization and treatment of waste salts, and a variety of technologies have been developed for the purification of waste salts, including pyrolysis technology (high-temperature melting) [10,11], washing [12,13], extraction [14,15], adsorption [16,17], and oxidation [18,19].

Pyrolysis technology holds promising application prospects in the field of waste salt treatment due to its advantages such as high organic matter removal efficiency and wide applicability. Its principle involves feeding waste salt into thermal equipment lined with refractory materials, followed by incineration or melting of the waste salt through flame or electric heating to achieve complete decomposition of organic matter and purification of salts [20], thereby realizing resource utilization of waste salts. However, during the melting or incineration process of waste salt, temperatures generally reach 800–1200 °C [9,21]. At this stage, a large amount of liquid phase is generated in the waste salt. These molten inorganic salts adhere to the surface of refractory materials and infiltrate into them, causing physical erosion and chemical corrosion to the refractory lining. This leads to refractory material spalling, compromises material performance, significantly shortens equipment lifespan, and increases operation and maintenance costs [22,23,24].

Refractory materials, as critical foundational materials in high-temperature industries, are widely applied as linings and protective layers in high-temperature industrial equipment [25,26]. Their key properties include corrosion resistance, thermal shock resistance, mechanical wear resistance, and low thermal conductivity, playing an irreplaceable role in the development of high-temperature industries. In high-temperature incinerators, the primary causes of refractory material damage include mechanical erosion and wear [27], infiltration of high-temperature melts and chemical reactions [28,29], as well as volatilization of high-temperature gas phases [30].

Magnesium-based refractory materials are widely utilized in high-alkaline environments such as steelmaking [31], metallurgy [32], and cement [33] industries due to their high melting point, excellent chemical stability, and superior resistance to alkaline medium corrosion. Extensive research has been conducted domestically and internationally to address the corrosion issues of magnesium-based refractories in high-alkaline environments. Zhang et al. [34] investigated the corrosion behavior of refractories during the preparation of Ti-Si-Al alloys via aluminothermic reduction of titanium-bearing blast furnace slag (TBFS). They found that MgO-based refractories exhibited optimal performance in TBFS aluminothermic reduction systems containing CaO alkaline slag. This was attributed to the spinel layer formed on the surface, which effectively inhibited slag infiltration and self-dissolution, resulting in significantly lower corrosion compared to Al_2_O_3_-based refractories. To enhance the alkali slag corrosion resistance of MgO-based refractories, Zhang et al. [35] proposed modifying the CaO/SiO_2_ mass ratio of the slag to improve the permeability and optimize the microstructure of magnesium-based refractories. Results demonstrated that the formation of new solid phases promoted the development of a dense isolation layer, preventing further slag infiltration. Cheng et al. [36] conducted systematic comparisons of the corrosion resistance of five refractory materials (MgO, MgO-Al_2_O_3_, MgO-Cr_2_O_3_, Al_2_O_3_ and Cr_2_O_3_) against typical sodium salts (NaCl, Na_2_CO_3_, Na_2_SO_4_) at temperatures ranging from 600 °C to 1200 °C through thermodynamic calculations (Factsage 7.2) and experimental validation. The study revealed that MgO refractories outperformed other materials in terms of corrosion resistance, thermal stability, and environmental friendliness due to their lower reactivity with sodium salts.

Previous studies have thoroughly confirmed the excellent corrosion resistance of magnesium-based refractories in traditional high-alkaline environments, establishing both theoretical and experimental foundations for their applications in steelmaking, metallurgy, and waste incineration. However, current domestic and international research on the corrosion of magnesium-based refractories by high-temperature molten pharmaceutical waste salt remains insufficient. Therefore, an in-depth investigation into the corrosion behavior of high-salt pharmaceutical waste liquid in its molten state against refractory materials—particularly exploring the service performance and failure mechanisms of MgO-based refractories under these specific conditions—holds significant theoretical value and engineering importance for developing efficient, long-lasting, and cost-effective incineration treatment systems. This paper aims to systematically analyze the corrosion mechanisms of refractory materials during pharmaceutical waste salt incineration, with a focus on examining the corrosion resistance characteristics of MgO-based refractories against pharmaceutical waste salt. This work provides a scientific basis for the optimal selection and design of lining materials for high-temperature pharmaceutical waste salt incineration equipment.

## 2. Materials and Methods

### 2.1. Material Preparation

#### 2.1.1. Preparation of Simulated Pharmaceutical Waste Salt

This study first performed XRF and XRD analyses on the waste salt produced by a pharmaceutical manufacturer in China to determine its chemical composition and phase types. The main elemental composition is shown in Table 1. It can be observed that the pharmaceutical waste salt contains high levels of Na, Cl, O, S, and Ca, along with other non-metallic and heavy metal elements.

**Figure 1:**
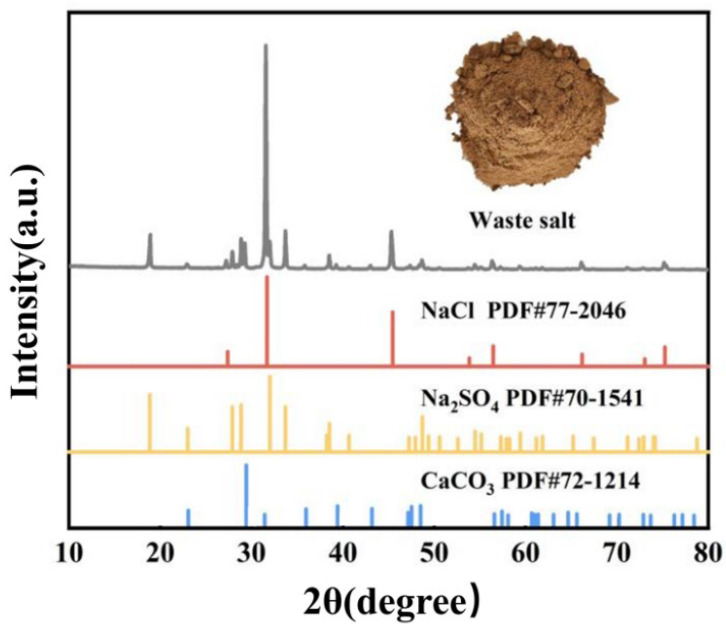
Physical image and XRD pattern of pharmaceutical waste salt sample.

Figure 1 shows the physical photograph and XRD pattern of the pharmaceutical waste salt sample. As can be seen from the physical photograph, the waste salt appears as light brown particles, which is due to the presence of elements such as Fe, Br, and Cr in the waste salt. Compounds formed by these three elements often exhibit brown, reddish-brown, and green colors, resulting in the light brown appearance of the waste salt. Analysis of the XRD test results of the waste salt showed that the main phase compositions are NaCl, Na_2_SO_4_, and CaCO_3_. Based on the XRD and XRF test results, the chemical composition of the pharmaceutical waste salt was calculated as 63.74 wt% NaCl, 26.19 wt% Na_2_SO_4_, 6.07 wt% CaCO_3_, and 4 wt% of other compounds.

Considering the complex chemical composition and certain variability in the components of the pharmaceutical waste salt, the experiment optimized the composition of the pharmaceutical waste salt and used chemical reagents (NaCl, Na_2_SO_4_, and CaCO_3_; Aladdin, Shanghai, China) to prepare a simulated mixed pharmaceutical waste salt with a composition of 65 wt% NaCl-30 wt% Na_2_SO_4_-5 wt% CaCO_3_.

#### 2.1.2. Preparation of Refractory Materials for Wetting and Corrosion Experiments

This experiment selected two types of magnesium-based refractory materials with different phase compositions produced by Beijing Ruitai Technology Co., Ltd. (Beijing, China) for corrosion experiments. These refractory materials were prepared by semi-dry pressing followed by high-temperature sintering. The specific compositions of the two refractory materials are shown in Table 2, with data sourced from the company’s product manual.

The refractory materials were cut into 20 × 30 × 3 mm thin slices for surface wettability testing. For corrosion experiments, cylindrical specimens (Φ12 mm × 70 mm) were extracted from the bulk refractory materials using a hollow diamond drill bit (inner diameter 13 mm, rotational speed 300 rpm) with continuous water cooling to prevent thermal damage. The drilled cores were then cut to the required length and ground to final dimensions.

### 2.2. Surface Wettability Experiment

**Figure 2:**
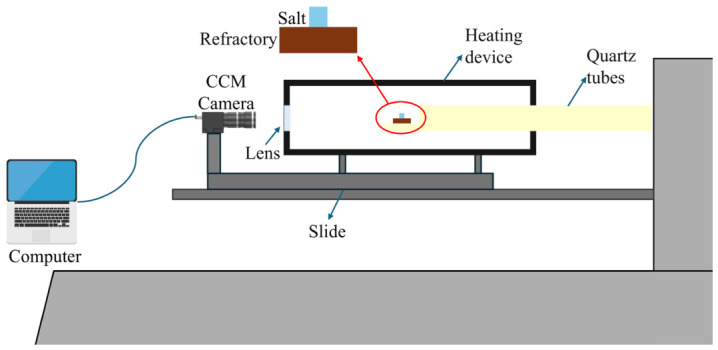
Schematic diagram of the wetting experimental apparatus.

The wetting and infiltration characteristics of pharmaceutical waste salt melt on refractory materials were measured using the sessile drop method. The pharmaceutical waste salt particles were ground to below 74 μm in diameter, pressed into a cylindrical shape with a diameter of 1 mm, and placed on top of the prepared refractory material thin slice. The sample was placed above a quartz tube and heated from room temperature to 1000 °C at 10 °C/min. Through a transparent window, a CCM camera was used to record to photograph the melting process, capturing the droplet shape every 5 s. Data was recorded via computer. The experimental schematic diagram is shown in Figure 2.

To more directly observe the wetting and infiltration interface of the molten salt on the refractory material surface, the refractory material thin slices after molten salt infiltration were cut from the middle. To prevent the removal of the infiltrated molten salt, the sample cross-sections were polished using a dry polishing method. Scanning electron microscopy (ZEISS Sigma 360, Oberkochen, Baden-Württemberg, Germany) was used at a voltage of 15 kV and 100× magnification to observe the microstructure of the refractory materials after infiltration. Energy dispersive spectroscopy (EDS) was used to analyze the infiltration of various elements at the refractory material interface.

### 2.3. Static Corrosion Experiment

The cylindrical refractory material (Φ12 mm × 70 mm) and the prepared mixed salt were placed together in a corundum crucible. After covering the crucible with a ceramic lid, the crucible was placed in a high-temperature (SJF1750 150 × 200 mm, Boyuntong Instrument Technology Co., Ltd., Nanjing, China) furnace and heated at a rate of 10 °C/min to 950 °C and 1150 °C respectively, with corrosion lasting for 48 h. During the holding period, simulated salt was added to the crucible every 1.5 h. After the holding period, the samples were removed, cooled to room temperature, and subjected to subsequent testing.

To more accurately determine the possible reaction phases and accelerate the reaction between refractory material and molten salt, the refractory material was ground into powder and mixed with the mixed salt at a mass ratio of 1:5. After mixing, it was placed in a corundum crucible and calcined at high temperatures of 950, 1050, 1150, 1250, and 1350 °C for 6 h.

### 2.4. Characterization of Corrosion Products

The corroded refractory materials were cut into 2 mm thin slices, polished, and then observed using scanning electron microscopy (ZEISS Sigma 360, Oberkochen, Baden-Württemberg, Germany) at 15 kV voltage with 50× and 100× magnification to examine changes in the microstructure of the refractory materials after corrosion. Energy dispersive spectroscopy (EDS) was used to analyze the distribution of elements at the interface of the refractory materials. The sintered samples were removed, ground into powder with particle diameter below 74 μm using an agate mortar, and subjected to X-ray diffraction analysis using a copper target at a scanning speed of 2°/min to investigate the phase changes in the refractory materials at different calcination temperatures.

### 2.5. Thermodynamic Calculation

The Equilib module in FactSage 8.2 (Thermochemical Software and Database, GTT-Technologies, Herzogenrath, Germany) was used to simulate the reaction process between molten salt and refractory materials. By inputting the composition of the mixed salt and refractory materials into the module, setting the calculation temperature range from 950 °C to 1350 °C with a step size of 25 °C, pressure at 1 atm, and selecting FactPS and FToxid databases. The types of phases generated when the mixed salt and refractory materials reach chemical equilibrium were obtained.

## 3. Results

### 3.1. Wetting and Infiltration Experimental Results

**Figure 3:**
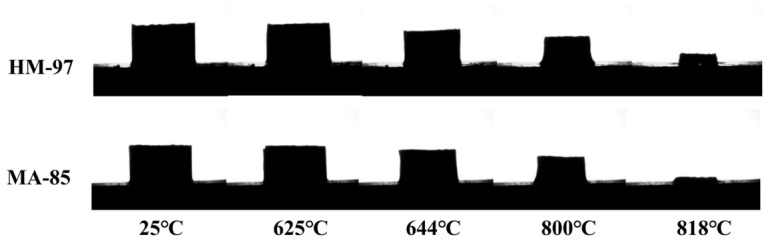
Wetting and infiltration process of the molten column on different refractory materials.

Figure 3 shows the high-temperature imaging of the wetting and infiltration process of the molten column on different refractory materials. It can be observed that the molten column undergoes volume contraction at around 625 °C, beginning to melt slowly. This is because the mixing of the three salts (NaCl, Na_2_SO_4_, and CaCO_3_) significantly lowers the melting point. After 818 °C, the remaining solid on the refractory material surface was high-melting-point CaO solid powder produced by the decomposition of CaCO_3_. The molten column hardly exhibits any wetting behavior on the two refractory materials, only infiltration behavior. This is because the molten salt has low viscosity and small surface tension at high temperatures, so it cannot form a hemispherical contour on the refractory material surface like glass melt or slag [37,38]. Moreover, MgO-based refractory materials have high porosity, leading to direct infiltration of the molten salt into the refractory material.

**Figure 4:**
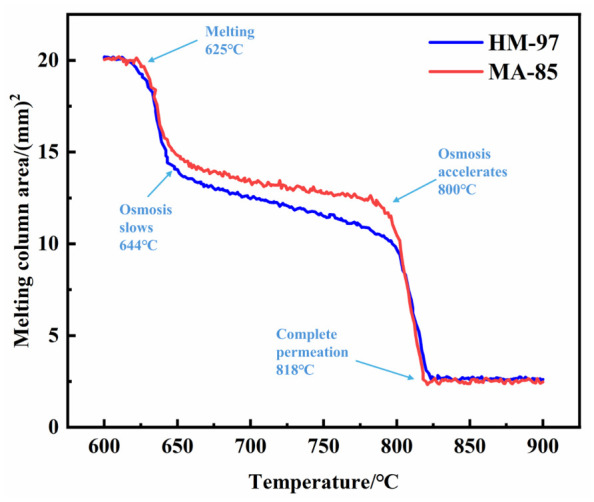
Curve of molten column shadow area variation with temperature.

Figure 4 shows the curve of the molten column shadow area changing with temperature. Combined with Figure 2 analysis, before 625 °C, the molten column does not melt, and the area remains unchanged. Between 625 °C and 644 °C, the area significantly decreases as the molten column begins to melt, with some molten salt infiltrating. From 644 °C to 800 °C, the area slowly decreases, with MA-85 samples showing significantly lower infiltration rates than HM-97 samples. Between 800 °C and 818 °C, the area rapidly decreases, likely because the molten column completely liquefies, viscosity significantly decreases, and it quickly infiltrates into the refractory material.

**Figure 5:**
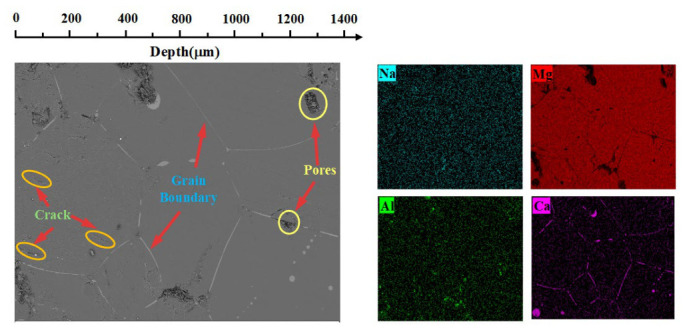
SEM-EDS images of cross-sections of HM-97 refractory material after wetting and infiltration experiments.

**Figure 6:**
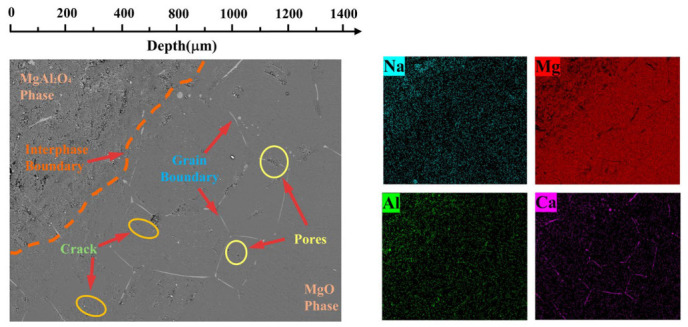
SEM-EDS images of cross-sections of MA-85 refractory material after wetting and infiltration experiments.

Figure 5 and Figure 6 show the SEM-EDS scanning images of the cross-sections of the two refractory materials after wetting and infiltration experiments. The apparent porosity of HM-97 and MA-85 refractory materials is ≤18% and ≤17%, respectively, as provided by the manufacturer (Table 2). However, significant differences exist in their pore structures and phase compositions. From the SEM images, it can be seen that the cross-sectional surfaces of both refractory materials contain numerous grain boundaries, pores, and microcracks. Notably, HM-97 exhibits a higher density of interconnected pores and finer grain structure, whereas MA-85 demonstrates a more heterogeneous microstructure with distinct phase boundaries.

In HM-97 refractory material, the fine periclase grains create abundant grain boundaries and interconnected pore channels. The microcracks exhibit a branch-like morphology, further enhancing connectivity between pores. EDS results indicate Ca element enrichment regions at these pores, suggesting that the interconnected pore structure may serve as rapid infiltration pathways for molten salt. The high density of grain boundaries in HM-97 also facilitates elemental diffusion along these interfaces.

In contrast, the MA-85 refractory material shows the MgAl_2_O_4_ phase in the upper left region, with a clear phase boundary observed. The MgAl_2_O_4_ grains are significantly coarser with fewer grain boundaries, and Ca elements barely infiltrate into this phase region. The isolated, non-connected pore structure in the spinel-rich regions provides fewer infiltration channels for the molten salt. Although the overall apparent porosity of MA-85 (≤17%) is comparable to or slightly lower than that of HM-97 (≤18%), the critical distinction lies in the pore connectivity and grain boundary density rather than total porosity alone.

In summary, the significantly lower infiltration rate of MA-85 samples compared to HM-97 between 644 °C and 800 °C can be attributed to the combined effects of microstructural and compositional differences. The interconnected pore structure in HM-97 enables rapid molten salt penetration, whereas the more isolated pores in MA-85 restrict fluid flow. Additionally, the fine periclase grains in HM-97 provide abundant grain boundaries that serve as diffusion pathways for elements, while the coarse MgAl_2_O_4_ grains in MA-85 minimize such channels. Furthermore, the MgAl_2_O_4_ spinel phase appears to exhibit resistance to molten salt infiltration, potentially blocking element penetration. These factors collectively govern the distinct wetting and infiltration behaviors observed in the two refractory materials.

### 3.2. Microstructure of Refractory Materials After Corrosion

**Figure 7:**
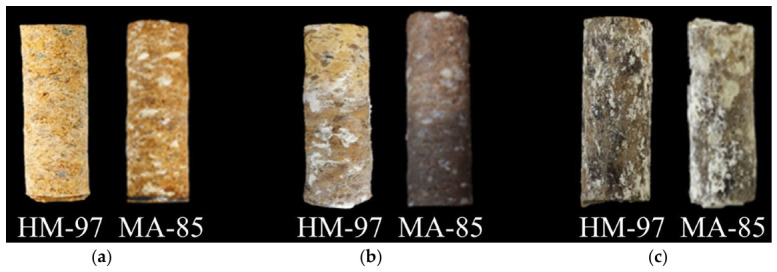
Physical images of refractory materials before and after corrosion. (**a**) Before corrosion; (**b**) after 950 °C/ 48 h corrosion; (**c**) after 1150 °C/48 h corrosion.

Figure 7a shows the physical photograph of the refractory materials before corrosion. It can be seen that both surfaces are relatively rough, with many pores. Compared to HM-97 refractory material, MA-85 refractory material has abundant white MgAl_2_O_4_ phase on its surface. Figure 7b shows the physical photographs of the two refractory materials after 48 h of corrosion at 950 °C. It can be seen that the HM-97 refractory material surface is attached with a large amount of white granular material, while the MA-85 refractory material surface does not show this. Figure 7c shows the physical photographs of the two refractory materials after 48 h of corrosion at 1150 °C. It was found that both surfaces produced a large amount of white granular attachments with a loose texture.

**Figure 8:**
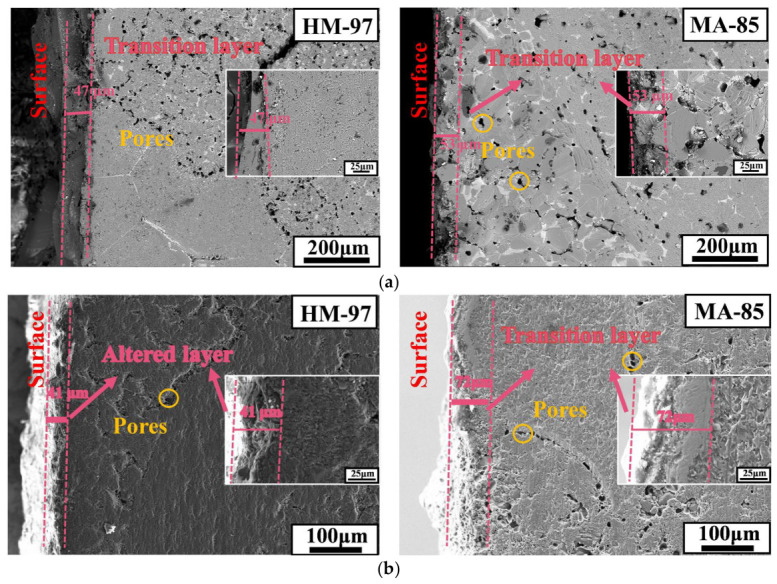
Morphology images of HM-97 (**left**) and MA-85 (**right**) before and after corrosion. (**a**) After 950 °C/ 48 h corrosion; (**b**) after 1150 °C/48 h corrosion.

Figure 8a shows the microstructures of refractory materials after 48 h of corrosion at 950 °C. After corrosion at 950 °C, the HM-97 sample developed a transition layer of approximately 47 μm with uneven corrosion morphology, while the MA-85 sample formed a transition layer of about 53 μm. Neither refractory material exhibited a significant metamorphic layer.

Figure 8b shows the microstructure images of HM-97 and MA-85 samples after corrosion at 1150 °C. The HM-97 sample developed long cracks and uneven corrosion morphology, but the transition layer thickness remained almost unchanged. The MA-85 sample’s transition layer thickness increased to approximately 72 μm, with a small amount of granular material attached to the transition layer, and a dense metamorphic layer was formed.

**Figure 9:**
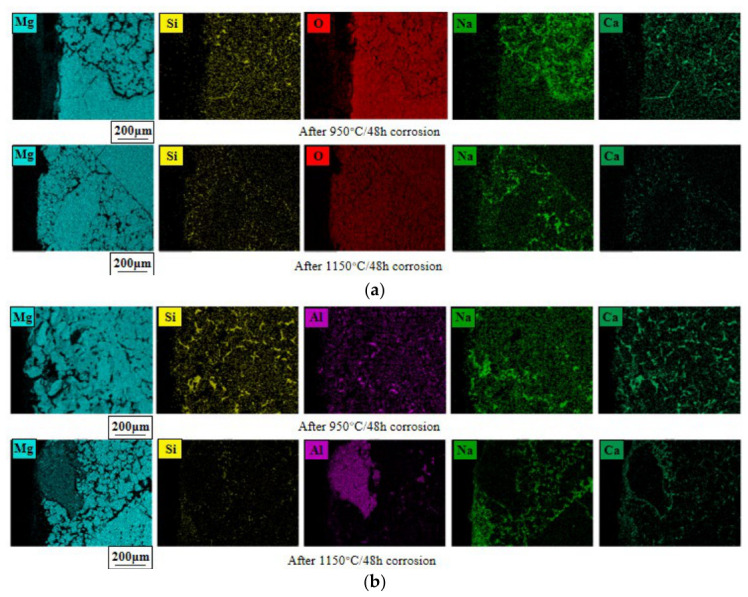
Post-corrosion EDS elemental mapping of magnesia-containing samples. (**a**) HM-97; (**b**) MA-85.

Figure 9a,b present a comparative analysis of the element distribution in HM-97 and MA-85 refractory materials after 48 h of molten salt corrosion at 950 °C and 1150 °C.

For HM-97 samples (Figure 9a): At 950 °C, Mg elements are uniformly distributed within the periclase phase. Si elements are diffusely distributed in the glass phase region, while Na and Ca elements are scattered and enriched at pores, cracks, and glass phase interfaces, with Na content higher than Ca, indicating that monovalent alkali metal ions diffuse more easily, and molten salt preferentially infiltrates through grain boundaries and defect channels. At 1150 °C, the overall concentration of Si and Ca elements decreases.

For MA-85 samples (Figure 9b): Al elements are mainly distributed in the magnesium–aluminum spinel phase. Na and Ca elements find it difficult to infiltrate into the MgAl_2_O_4_ phase, which effectively prevents molten salt infiltration, further confirming the results of the wetting and infiltration experiments in Section 2.1. At 950 °C, Si elements are enriched in the glass phase, while Na and Ca elements are enriched at cracks, holes, and MgO-MgAl_2_O_4_ phase interfaces. At 1150 °C, Si and Ca elements, like in HM-97 samples, decrease overall.

### 3.3. Phase Analysis Before and After Corrosion

**Figure 10:**
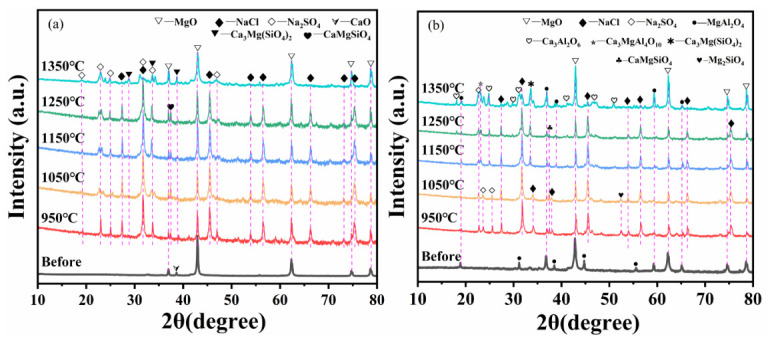
XRD pattern of magnesia-containing bricks before and after corrosion. (**a**) HM-97; (**b**) MA-85.

Figure 10a shows the XRD patterns of HM-97 refractory material powder before and after calcination with mixed salt. It can be seen that at 950 °C, some forsterite phase (CaMgSiO_4_) and Ca_3_Mg(SiO_4_)_2_ phase (merwinite-type) were identified in HM-97. As the temperature continued to increase, the forsterite phase (CaMgSiO_4_) in HM-97 appeared to transform into the Ca_3_Mg(SiO_4_)_2_ phase.

Figure 10b shows the XRD patterns of MA-85 refractory material powder before and after calcination with mixed salt. It can be seen that in MA-85, two different forsterite phases (Mg_2_SiO_4_ and CaMgSiO_4_) were identified at 950 °C. At 1150 °C, Ca_3_Mg(SiO_4_)_2_ phase was detected, and a small amount of MgAl_2_O_4_ phase (spinel solid solution) appeared to form in situ. At 1350 °C, some Ca_3_Al_2_O_6_ slag phase was detected.

Further XRD analysis, although qualitative, suggests that the white granular material observed on the surface of HM-97 samples at 950 °C in the physical diagram in Section 2.2 is consistent with Ca_3_Mg(SiO_4_)_2_, and the uneven corrosion morphology on the surface is likely related to the dissolution of Ca_3_Mg(SiO_4_)_2_ phase into the molten salt. At 1150 °C, white granular material was produced on the surface of both refractory materials, and certain corrosion morphology was observed. We speculate that the Ca_3_Mg(SiO_4_)_2_ phase present in HM-97 at 1150 °C may have dissolved into the mixed salt, potentially contributing to a decrease in the thickness of the refractory material, which may explain why the transition layer remained almost unchanged. The dense layer produced in MA-85 at 1150 °C in Figure 8b might be the in situ generated MgAl_2_O_4_ phase (spinel solid solution). The overall decrease in Si and Ca elements in both refractory materials at 1150 °C in the EDS diagram in Section 2.2 is likely attributed to the formation of Ca_3_Mg(SiO_4_)_2_ phase and its subsequent dissolution into the molten salt, as inferred from the qualitative phase analysis.

### 3.4. Thermodynamic Equilibrium Calculation

**Figure 11:**
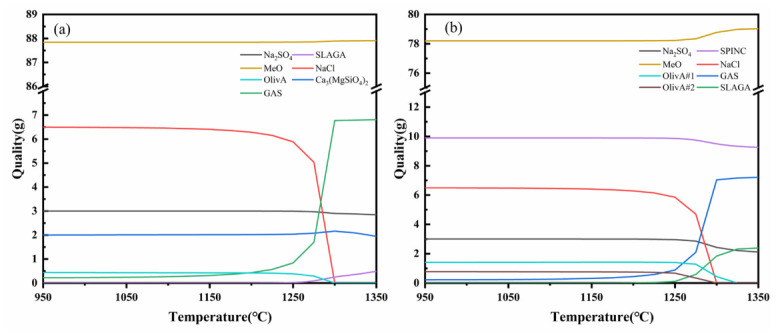
Thermodynamic equilibrium calculation of magnesium-containing refractory materials. (**a**) HM-97; (**b**) MA-85.

Figure 11 shows the equilibrium process and final products of the reaction between molten salt and refractory materials for HM-97 and MA-85 using the Equilib module in FactSage 8.2.

Figure 11a shows the reaction equilibrium process for HM-97. The image indicates that at 950 °C, the reaction system contains components such as OlivA (forsterite phase), Ca_3_Mg(SiO_4_)_2_, Na_2_SO_4_, NaCl, GAS (gas phase), and MeO (MgO, CaO). The initial GAS component is CO_2_ produced by the decomposition of CaCO_3_. As the temperature increases, at 1150 °C, NaCl begins to volatilize to form a gas phase, and the GAS component mass increases. At 1300 °C, NaCl completely volatilizes, corresponding to the disappearance of the NaCl phase in the XRD at 1350 °C in Figure 10a. At 1250 °C, part of the OlivA component generates Ca_3_Mg(SiO_4_)_2_. Another part dissolves into SLAGA (molten salt), corresponding to the disappearing of the CaMgSiO_4_ phase in the XRD at 1350 °C in Figure 10a, and many miscellaneous peaks appear at 1350 °C, which are speculated to be the generated slag phase.

Figure 11b shows the reaction equilibrium process for MA-85. The image indicates that at 950 °C, the reaction system contains components such as OlivA #1 (forsterite phase #1), OlivA #2 (forsterite phase #2), Na_2_SO_4_, NaCl, GAS (gas phase), SPINC (MgAl_2_O_4_ spinel phase), and MeO (MgO, CaO). The initial GAS component is CO_2_ produced by the decomposition of CaCO_3_. As the temperature increases, similar to HM-97, the NaCl peak in the XRD vanishes at 1350 °C, and the two forsterite phases dissolve into SLAGA at 1300 °C and 1325 °C respectively, corresponding to the disappearing of the two forsterite phases in the XRD at 1350 °C in Figure 10b. After 1250 °C, SPINC begins to dissolve into the molten salt, producing slag phase, corresponding to the generation of a large number of Ca_3_Al_2_O_6_ peaks in the XRD at 1350 °C in Figure 10b.

Through thermodynamic equilibrium calculation, it is concluded that Na salt hardly participates in the reaction in the system. HM-97 is more prone to react with the molten salt to generate Ca_3_Mg(SiO_4_)_2_, leading to the corrosion of the refractory material, while the MgAl_2_O_4_ spinel phase remains stable at 1300 °C, exhibiting strong corrosion resistance and dissolution resistance.

## 4. Discussion

This study reveals the corrosion mechanisms of MgO-based refractory materials in a molten pharmaceutical waste salt environment. By comparative analysis of high-purity MgO refractory material (HM-97) and magnesium–aluminum spinel composite refractory material (MA-85), the fundamental relationship between microstructure characteristics, phase stability, and corrosion resistance is revealed.

### 4.1. Effect of Microstructure on Molten Salt Infiltration

Wetting and infiltration experiments revealed significant temperature-dependent characteristics in molten salt infiltration. In the 625–644 °C range, the shadow area of the molten column rapidly decreased due to initial melting and gravity-driven infiltration through capillary action into open pores and microcracks. As the surface pores became gradually filled, the infiltration rate decreased. In the 644–800 °C range, the infiltration rate significantly decreased and stabilized. This can be attributed to three factors: the high viscosity of molten salt near the NaCl-Na_2_SO_4_-CaCO_3_ eutectic point, the filling of surface pores which weakened capillary action, and the establishment of dynamic equilibrium as infiltration was primarily governed by physical factors. In the 800–818 °C range, the shadow area decreased rapidly again due to CaCO_3_ decomposition. The release of CO_2_ gas created escape channels, while the reaction between produced CaO and molten salt formed low-viscosity silicate melts, collectively accelerating infiltration. The differences between HM-97 and MA-85 materials were also related to their phase stability at elevated temperatures. The MgAl_2_O_4_ spinel phase in MA-85 maintained structural integrity without significant interfacial reactions, whereas the fine-grained periclase in HM-97 provided more infiltration channels during the initial stage. The coarse-grained MgAl_2_O_4_ phase appeared to suppress infiltration through reduced grain boundaries and potentially improved phase interface stability.

### 4.2. Effect of Temperature on Corrosion Process and Dense Layer Formation

At 950 °C, both refractory materials formed transition layers (HM-97: 47 μm, MA-85: 53 μm) without noticeable metamorphic layers (Figure 8a). The uneven erosion morphology of HM-97 is likely attributed to the dissolution of Ca_3_Mg(SiO_4_)_2_ (calcium magnesium olivine) into the molten salt, as supported by qualitative XRD analysis (Figure 10a). This phase is proposed to correspond to the white granular material observed in Figure 7b, potentially formed through reactions between MgO, SiO_2_, and CaO (derived from the decomposition of CaCO_3_ in the molten salt).

**Figure 12:**
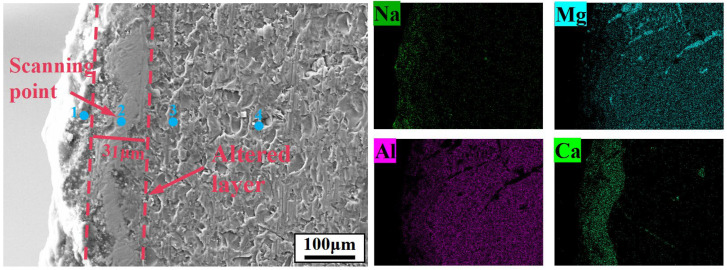
EDS element scanning of the dense layer produced in the MA-85 sample at 1150 °C.

At 1150 °C, HM-97 exhibited extended cracks, but the transition layer thickness remained nearly unchanged or slightly decreased (Figure 8b). The decrease in Si/Ca concentration in the EDS maps (Figure 9a) suggests that the dissolution of olivine into the slag phase may have been dominant, potentially leading to material loss rather than an increase in transition layer thickness. In contrast, MA-85 formed a 72 μm thick transition layer accompanied by a dense metamorphic layer (Figure 8b). To identify the main components of the metamorphic layer, EDS point scanning was performed at four representative locations within and around the dense layer (Figure 12, Table 3). Points 3 and 4, located in the interior of the dense layer, show high Mg and Al mass fractions (16.98 wt% and 35.96 wt% at point 3; 16.89 wt% and 36.12 wt% at point 4), corresponding to an atomic ratio approaching 1:2, together with negligible Na and Ca and extremely low Si and Cl. This indicates that the main component is MgAl_2_O_4_ (spinel solid solution). Points 1 and 2, situated near the surface or at the transition layer boundary, exhibit higher Na, Ca, and Cl contents, confirming active infiltration at these regions. The surface scanning results (Figure 12) further demonstrate that Na and Ca elements are effectively isolated outside the protective layer, while the point analysis provides direct compositional evidence that the dense layer is predominantly MgAl_2_O_4_-based, consistent with its potential to resist further infiltration of the molten salt.

The thermodynamic calculations (Figure 11) and experimental results are consistent with the interpretation that MgAl_2_O_4_ in MA-85 likely exhibits excellent stability. The continuous presence of the spinel peak in the XRD pattern up to 1350 °C (Figure 10b) confirms its thermal stability. This stability prevents further infiltration of the molten salt, effectively isolating the base refractory material from further erosion.

In contrast, HM-97 lacks a protective spinel phase, which may contribute to continuous degradation. At high temperatures (Figure 10a), the continuous formation and dissolution of calcium magnesium olivine (Ca_3_Mg(SiO_4_)_2_) create a cyclic process: (1) salt infiltration along grain boundaries, (2) reaction to form low-melting-point silicates, (3) dissolution of these phases into the molten salt, and (4) further infiltration. This proposed mechanism provides a plausible explanation for the significant surface erosion of HM-97 at 1150 °C, while the transition layer thickness appears to remain almost unchanged.

### 4.3. Corrosion Mechanism of Pharmaceutical Waste Salt on Refractory Materials

Based on the above discussion, the HM-97 and MA-85 refractory materials exhibit two completely different wetting behaviors, infiltration mechanisms, and reaction mechanisms. Therefore, they also present different erosion mechanisms.

**Figure 13:**
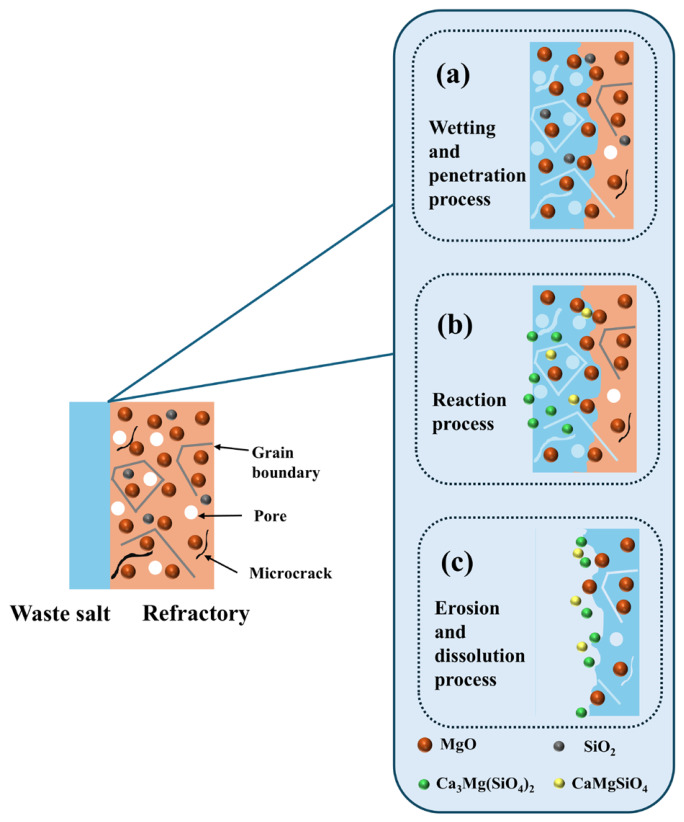
Schematic diagram of the corrosion mechanism of pharmaceutical waste salt on HM-97 refractory material.

Figure 13 is a schematic diagram of the erosion mechanism of the HM-97 refractory material. As shown in the figure, during the wetting and infiltration process, the molten pharmaceutical waste salt penetrates into the interior of the material through the pores, grain boundaries, and microcracks within the refractory material. After the wetting and infiltration process is complete, the MgO and SiO_2_ within the refractory material react with the CaO in the molten pharmaceutical waste salt to form low-melting-point silicates such as CaMgSiO_4_ and Ca_3_Mg(SiO_4_)_2_ (reactions (1) and (2)). Subsequently, the CaMgSiO_4_ and Ca_3_Mg(SiO_4_)_2_ generated by these reactions continuously dissolve into the slag, causing erosion of the refractory material. The molten pharmaceutical waste salt continues to deepen its infiltration, forming a cyclic process of infiltration–reaction–dissolution.

**Figure 14:**
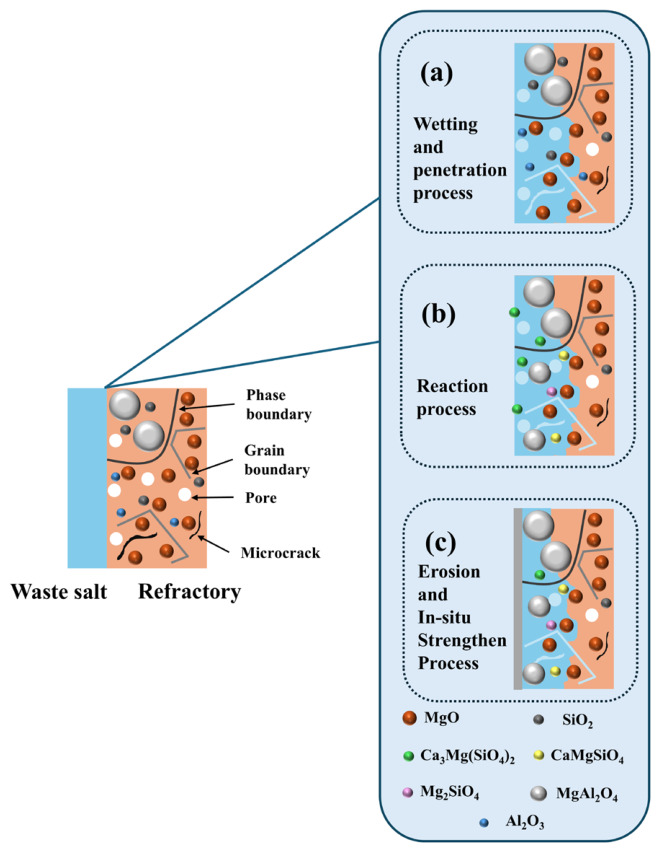
Schematic diagram of the corrosion mechanism of pharmaceutical waste salt on MA-85 refractory material.

As shown in Figure 14, during the wetting and infiltration process, similar to the HM-97 refractory material, the molten pharmaceutical waste salt penetrates into the interior of the material through the pores, grain boundaries, and microcracks within the MA-85 refractory material. However, the spinel phase region has fewer grain boundaries, which hinders the further infiltration of the molten salt into the refractory material.

After the wetting and infiltration process is complete, it is proposed that the MgO and SiO_2_ within the refractory material may react with the CaO in the molten pharmaceutical waste salt to form low-melting-point silicates such as CaMgSiO_4_ and Ca_3_Mg(SiO_4_)_2_ (reactions (1) and (2)). Simultaneously, the MgO, SiO_2_, and Al_2_O_3_ within the refractory material in situ react to form MgAl_2_O_4_ and Mg_2_SiO_4_ (reactions (3) and (4)).

As MgAl_2_O_4_ appears to continue forming, a dense metamorphic layer is proposed to be created on the surface of the refractory material, potentially preventing further infiltration of the molten pharmaceutical waste salt.

Reaction (1): CaO + MgO + SiO_2_ → CaMgSiO_4_

Reaction (2): 3CaO + MgO + 2SiO_2_ → Ca_3_Mg(SiO_4_)_2_

Reaction (1): CaO + MgO + SiO_2_ → CaMgSiO_4_

Reaction (2): 3CaO + MgO + 2SiO_2_ → Ca_3_Mg(SiO_4_)_2_

Reaction (3): MgO + Al_2_O_3_ → MgAl_2_O_4_

Reaction (4): 2MgO + SiO_2_ → Mg_2_SiO_4_

## 5. Conclusions

(1) The primary crystal phase in HM-97 is fine-grained periclase (MgO), which is rich in grain boundaries internally. In contrast, the primary crystal phase in MA-85 is also periclase (MgO), but it additionally contains larger-grained magnesium–aluminum spinel (MgAl_2_O_4_) phase, resulting in fewer grain boundaries within the material. Consequently, during the temperature range of 644 °C to 800 °C, the infiltration rate of molten salt on the surface of HM-97 refractory material is greater than that of MA-85 refractory material.

(2) Temperature has a significant effect on the erosion behavior. After erosion at 950 °C, HM-97 and MA-85 form transition layers of 47 μm and 53 μm, respectively. At this temperature, white granular substances appear on the surface of HM-97, which is likely related to the formation and dissolution of the Ca_3_Mg(SiO_4_)_2_ phase. At 1150 °C, HM-97 develops extended cracks, and the transition layer thickness remains almost unchanged, but it is speculated that this is caused by the continuous dissolution of the refractory material into the molten salt. Although the transition layer of MA-85 thickens to about 72 μm, it forms a dense metamorphic layer that hinders further infiltration of the molten salt.

(3) Phase evolution analysis suggests that the HM-97 material appears more prone to react with the molten salt at high temperatures, potentially forming the Ca_3_Mg(SiO_4_)_2_ phase, which may lead to the destruction of the material structure. In contrast, the MgAl_2_O_4_ phase in the MA-85 material appears to be stable at high temperatures and may effectively hinder the infiltration of molten salt, thereby contributing to the material’s anti-erosion performance.

(4) Thermodynamic calculations and experimental results are mutually consistent with the interpretation that the MgAl_2_O_4_ phase likely possesses excellent resistance to salt erosion. Its apparent stability in high-temperature molten salt environments is proposed as a contributing factor to the superior anti-erosion performance of the MA-85 material.

## Figures and Tables

**Table 1:** Elemental composition of pharmaceutical waste salt sample.

Element	O	Na	S	Cl	Al	Si
Mass fraction/%	17.72	33.54	5.90	38.68	0.02	0.09
**Element**	**P**	**Br**	**Ca**	**Fe**	**Cr**	**Other**
Mass fraction/%	0.02	0.30	2.43	0.89	0.04	0.17

**Table 2:** Chemical composition and phase composition of refractory materials.

Samples	Chemical Composition			
	MgO	SiO_2_	Al_2_O_3_	Other
HM-97	96.5%	1%	-	2.5%
MA-85	85%	1%	8%	6%
**Samples**	Phase composition			Apparent Porosity
	Fayalite	Magnesium–aluminum spinel phase	Glass phase	-
HM-97	96.5%	-	3.5%	≤18%
MA-85	88%	11%	1%	≤17%

**Table 3:** Element content of point scanning in the dense layer of MA-85 sample.

Point	Elemental Mass Fraction (wt%)						
	O	Na	Mg	Al	Si	Cl	Ca
1	34.58	10.69	3.7	17.56	0.27	11.35	20.35
2	44.37	2.32	10.6	21.75	0.07	0.05	20.83
3	46.79	0.07	16.98	35.96	0.08	0	0.11
4	46.89	0.08	16.89	36.12	0	0.02	0

## Data Availability

The original contributions presented in this study are included in the article. Further inquiries can be directed to the corresponding author.

## Abbreviations

The following abbreviations are used in this manuscript:

TBFS	Titanium-bearing Blast Furnace Slag
XRD	X-ray Diffraction
XRF	X-ray Fluorescence
EDS	Energy Dispersive Spectroscopy
SEM	Scanning Electron Microscopy
CCM	CrashCam Mini
SLAGA	Slag Phase A
OlivA	Forsterite Phase
GAS	Gas Phase
SPINC	Spinel Phase
MeO	Metal Oxide
